# The assessment of procedural skills in physiotherapy education: a measurement study using the Rasch model

**DOI:** 10.1186/s40945-020-00080-0

**Published:** 2020-05-25

**Authors:** Karl Martin Sattelmayer, Kavi C. Jagadamma, Franziska Sattelmayer, Roger Hilfiker, Gillian Baer

**Affiliations:** 1 School of Health Sciences, Physiotherapy, Queen Margaret University, Edinburgh, Scotland; 2 School of Health Sciences, University of Applied Sciences and Arts Western Switzerland Valais (HES-SO Valais-Wallis), Leukerbad, Switzerland; 3 Berchtesgaden, Germany

**Keywords:** Motor skills [F02.808.260], Clinical competence [N05.715.175], Educational measurement [I02.399], Psychomotor performance [F02.808], Public health professional education [I02.358.556]

## Abstract

**Background:**

Procedural skills are a key element in the training of future physiotherapists. Procedural skills relate to the acquisition of appropriate motor skills, which allow the safe application of clinical procedures to patients. In order to evaluate procedural skills in physiotherapy education validated assessment instruments are required. Recently the assessment of procedural skills in physiotherapy education (APSPT) tool was developed. The overall aim of this study was to establish the structural validity of the APSPT. In order to do this the following objectives were examined: i) the fit of the items of APSPT to the Rasch-model, ii) the fit of the overall score to the Rasch model, iii) the difficulty of each test item and iv) whether the difficulty levels of the individual test items cover the whole capacity spectrum of students in pre-registration physiotherapy education.

**Methods:**

For this observational cross-sectional measurement properties study a convenience sample of 69 undergraduate pre-registration physiotherapy students of the HES-SO Valais-Wallis was recruited. Participants were instructed to perform a task procedure on a simulated patient. The performance was evaluated with the APSPT. A conditional maximum likelihood approach was used to estimate the parameters of a partial credit model for polytomous item responses. Item fit, ordering of thresholds, targeting and goodness of fit to the Rasch model was assessed.

**Results:**

Item fit statistics showed that 25 items of the APSPT showed adequate fit to the Rasch model. Disordering of item thresholds did not occur and the targeting of the APSPT was adequate to measure the abilities of the included participants. Undimensionality and subgroup homogeneity were confirmed.

**Conclusion:**

This study presented evidence for the structural validity of the APSPT. Undimensionality of the APSPT was confirmed and therefore presents evidence that the latent dimension of procedural skills in physiotherapy education consists of several subcategories. However, the results should be interpreted with caution given the small sample size.

## Introduction

Procedural skills are a key element in the training of future physiotherapists [1] and of other health professions [2]. Frequently, procedural skills are defined based on a definition by Kent as: “a skill involving a series of discrete responses each of which must be performed at the appropriate time in the appropriate sequence” [3]. This definition focuses mainly on the movement and biomechanical aspects of procedural skills. However, in physiotherapy education other aspects of procedural skills might be equally important. Therefore, a definition integrating more information was necessary for this study and procedural skills were operationalised as following. Procedural skills relate to the acquisition of appropriate motor skills, which allow the safe application of clinical procedures to patients. To adequately perform these skills, knowledge about manual or technical procedures must be acquired. Procedural skills may involve decision-making (i.e. selection of appropriate procedures) and communication processes (i.e. communication with the patient about the nature of the procedure). When procedures are actively performed in combination with patients (e.g. procedures in physiotherapy) patient-focussed interaction abilities are required.

Procedural skills in physiotherapy education relate to the execution of a practical task such as performing a soft tissue mobilisation or teaching a person with a stroke to perform a safe transfer to ground. A procedure can be related to a diagnostic intervention or to a therapeutic intervention. Incorrectly performed procedures may result in ineffective treatments or serious problems and adverse events to patients and health professionals. For example, in a recent systematic review, which included 368 studies, Gorrel and colleagues [4] reported mild adverse events following spinal manipulation in 61 studies and major adverse events in 2 studies. Anecdotally it is known that sometimes physiotherapist perform practical procedures in clinical situations with poor working positions, which might cause incorrect application of the procedure or musculoskeletal injuries [5]. Glista and co-workers [6] reported a considerable worsening of posture was observed in physiotherapy students during their study examining posture at the beginning and the end of a physiotherapy degree programme.

Several systematic reviews have been published reporting about measurement properties for procedural skills in health professions education [7–11]. Most of the reviews identified several assessment tools for procedural skills in medical education. For example, Jelovsek et al. [8] reported that over 30 tools are available in medical education and most of them are designed to measure procedural skills in surgical education. In general, the reviews identified that there is a lack of assessments for procedural skills in allied health professions. In a systematic review Sattelmayer and colleagues evaluated assessment tools in physiotherapy education [11]; the authors reported on the measurement properties of eight assessments. Six procedure specific assessments were identified (i.e. they can only be used to assess one specific procedure) and could only be used in the field of musculoskeletal practice. Two generic assessment tools were identified. Generic assessment of procedural skills are measurement instruments, which are applicable to a broad range of clinical procedure [9]. However, both assessments were not validated in the field of physiotherapy education. Therefore, there is a need to design valid generic assessments targeted to measure the broad spectrum of physiotherapeutic practice (e.g. neurological and respiratory practice).

To answer this need, the assessment of procedural skills in physiotherapy education (APSPT) was developed [12]. The APSPT is a generic assessment tool for procedural skills with 29 items. The APSPT contains six sub-categories (i.e. preparation, knowledge and decision-making, communication, safety, procedure execution and comfort). Each sub-category is evaluated with several specific items. For, example there are four specific items in the sub-category “preparation”. The outcome for each item ranges from “very poor” (0 points) to “very good” (4 points). In addition, the evaluator can check that a specific item was not assessed. Furthermore, each sub-category is evaluated with an overall assessment of this sub-category (e.g. “overall assessment preparation”). The scoring of the overall items is based on the evaluation of the specific items (i.e. the educator scores the specific items in advance). The APSPT with 29 items is presented in Additional file 1.

The scale was developed over three steps: i) a systematic review identified and appraised existing measurement instruments for procedural skills in physiotherapy education [11], ii) interviews with stakeholders (i.e. educators and students) were performed to discuss potentially relevant items and iii) a pilot study with 30 students was performed to analyse the feasibility and internal consistency of the scale [12]. Interrater reliability of the APSPT total score was adequate with an ICC of 0.79 [13]. However, the use of a summary score is only valid if the individual items of the assessment refer to the same dimension “procedural skills” [14]. Furthermore, it should be assessed whether the 29 individual items of the APSPT refer to same latent dimension. One way to assess this is with Rasch analysis [15]. Rasch analysis is a method from the field of item response theory. In item response theory the measurement properties of an instrument are evaluated on item and person level [16]. The person level refers to student’s ability or skill level and the item level provides information about the measurement properties of each individual item.

The overall aim of this study was to establish the structural validity of the APSPT in undergraduate education. In order to do this the following objectives were examined: i) the fit of the items of the APSPT to the Rasch-model (i.e. each individual test item of the APSPT should provide information about the latent dimension (“procedural skills”)), ii) the fit of the overall score to the Rasch model, iii) the difficulty of each test item and iv) whether the difficulty levels of the individual test items cover the whole capacity spectrum of students in pre-registration physiotherapy education.

## Methods

### Design

This observational cross-sectional measurement properties study was conducted in 2017.

### Participants

Data from a previous study in physiotherapy education were used for this study [13]. The study received approval from the ethical committee of Queen Margaret University (2017-02-17) and the Commission cantonale d'éthique de la recherche sur l'être humain (CER-VD) Switzerland (2016-12-08). A convenience sample of 69 undergraduate pre-registration physiotherapy students of the University of Applied Sciences Western Switzerland, Valais (HES-SO Valais-Wallis) (a single physiotherapy school) was recruited for this measurement properties study. All participants were members of a single physiotherapy school the HES-SO Valais-Wallis. The physiotherapy degree programme at the HES-SO Valais-Wallis consists of a 3-year undergraduate bachelor programme with a study load of 180 ECTS and each academic year has a capacity of 40 students. After successfully completing the 180 ECTS, students can register as physiotherapists in Switzerland. Students in the second and third year of their undergraduate education (i.e. within the bachelor programme) were eligible for inclusion. Within this study all participants were within their bachelor education and hence classified as “preregistration”. All participants had received formal training at the HES-SO Valais-Wallis regarding the procedural skills before this study. Participants performing the first task procedure were third year students and participants performing the second task procedure were second year students. The experience with the procedures was made within the university (i.e. trained in simulation and with peer students) and to our knowledge no prior experience existed regarding the use of the procedures within a clinical setting (i.e. with “real” patients).

Potential participants were approached by a research assistant and informed about the study. The participants had to provide written informed consent in case of study participation. Participants did not receive study credits for participation. All participants who fulfilled the inclusion criteria and volunteered to participate were included.

### Assessment of procedural skills in physiotherapy education

Participants were instructed to perform a task procedure on a simulated patient. The performance was video recorded and an independent rater evaluated the performance on the video recordings. Two different task procedures were evaluated. The first task procedure was a transfer to the ground for a person with a stroke and the second task procedure was a set of procedures from vestibular rehabilitation. These procedures were the Dix Hallpike test [17], the Liberartory manoeuvre [18] and the Canalith repositioning technique [19]. The performance of the participants was evaluated with the APSPT with 29 items.

### Overview of the analyses

The statistical analysis was performed using the statistical software package R [20] and the user written packages eRm [21], psych [22] and Gifi [23]. A conditional maximum likelihood approach [24] was used to estimate the parameters of a partial credit model for polytomous item responses [25]. The estimations of the test difficulty and the ability of the participants are reported in logit units (i.d. log of the odds). Higher logits indicate higher difficulty of the test or higher ability of the person. In addition, logit units were transformed to a 0–100 score [26]. The lowest logit score was set equal to 0 and the highest logit score was set equal to 100 [27, 28]. Person and items are still scored on the same continuum and can be compared with each other. The advantage of the 0–100 scale is that only positive numbers are used, which might be more straightforward for the interpretation of the study data.

### Sample size

The sample size of this study was based on recommendations of Linacre [29]. To receive stable item calibrations or person measures within ½ logits the minimum sample size was set to 64 participants.

### Item fit

Four test statistics were used to evaluate the item fit of each test item. Outfit and infit mean-square values and outfit and infit standardised t-values were used. Item fit was assessed using a classification presented by Linacre [30]. Mean-square statistic values between 0.5 and 1.5 were classified as productive for the measurement. A range between − 2 and 2 for standardised t-values was used to indicate acceptable item fit. Values below the acceptable range of fit were classified as overfit and values above were categorised as underfit.

### Threshold disordering and targeting

All items were evaluated regarding threshold disordering. It was expected that the difficulties of the thresholds should increase with increasing thresholds (i.e. the logit value of threshold 1 should be less difficult than the logit value of threshold 2). Furthermore, it was evaluated whether the targeting of the range of the items on the latent dimension (procedural skills) was adequate to measure the abilities of the participants. That is, most of the participants should be located within the range of item estimates [31]. This was analysed using a person item map.

### Goodness of fit of the Rasch model

The goodness of fit of the Rasch model was evaluated with two likelihood ratio (LR) tests [32]. The likelihood ratio test is based on the assumption of subgroup homogeneity. That is estimated parameters should be equal between subgroups. For this study the subgroup homogeneity was assessed for groups performing the different procedures (i.e. the transfer and the vestibular rehabilitation procedure). A second LR test has been run using the mean score as split criterion (i.e. estimated parameters were compared for participants scoring below the mean and above the mean). To test the assumption of undimensionality three tests were used. First, the Martin Löf test [33] and an exact version of the Martin Löf test were used [34]. In order to apply the exact version, the data of the APSPT were dichotomised (i.e. the test is designed for binary data matrices). Response categories were collapsed into two categories: “very poor” and “poor” performances and “adequate”, “good” and “very good” performances were combined. Third, a categorical principal component analysis was used to assess undimensionality of the APSPT visually. Therefore, a two-dimensional Princals solution using the Gifi package was applied [23]. To enhance reporting procedures, a modified version of the STROBE checklist for observational studies [35] was used (Additional file 2).

## Results

### Overview of the sample

In total 79 students were asked to participate in this study and 69 fulfilled the inclusion criteria, agreed to participate and were included in this study. The most frequent reason for non-participation were time constraints.

The mean age of the included participants was 24.1 years (SD: 1.9). Considerably more female participants volunteered for this experiment and were recruited (i.e. 53 female versus 16 male participants). For the task procedure “transfer” 31 participants volunteered and were recruited and 38 participants performed task procedure 2 (i.e. the set of procedures from vestibular rehabilitation). The groups performing the different task procedures were similar regarding demographic data (Table 1). Previous academic performance regarding examinations of procedural skills measured on a scale ranging between 0 (low) and 6 (high) was comparable between groups.

**Table 1:** Demographic data of included participants

	Transfer (*N* = 31)	Vestibular Rehabilitation (*N* = 38)	Total (*N* = 69)
**Sex**			
Female	23 (74.2%)	30 (78.9%)	53 (76.8%)
Male	8 (25.8%)	8 (21.1%)	16 (23.2%)
**Age years**			
Mean (SD)	23.1 (1.3)	24.9 (1.9)	24.1 (1.9)
Range	21–26	23–32	21–32
**Previous academic performance (0–6)**			
Mean (SD)	5.1 (0.3)	5.0 (0.3)	5 (0.3)
Range	4.4–5.6	4.1–5.5	4.1–5.6
Training level (year of training)	3	2	

NB. Previous academic performance in the Swiss education system can range between 0 (low) and 6 (high)

### Statistical model

The partial credit model estimation showed a conditional log-likelihood of − 960.99. The parameter estimation converged after 66 iterations and 86 parameters were estimated.

### Item fit

**Fig. 1:**
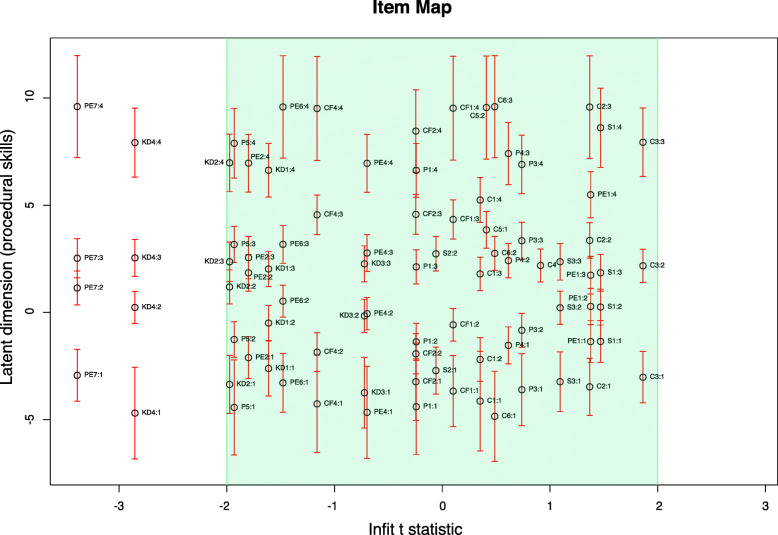
Bond and Fox pathway map. The location of each item is plotted against its infit t-statistic. The infit t-statistic should be between 2 and − 2 (green area). Points indicate the item difficulty and the corresponding 95%CIs are plotted vertically. NB. The item fit is presented for all item thresholds. For example, thresholds 1, 2, 3 or 4 of item PE7 can be identified as PE7:1, PE7:2, PE7:3 and PE7:4

Item fit was analysed with outfit and infit mean-square and outfit and infit standardised t-values. During initial testing several items were identified with item misfit. First, item PE5 (uninterrupted flow of the procedure) showed misfit (underfit) on all four item fit statistics. Second, item P2 (checks and prepares environment) showed misfit with regard to outfit mean-square and outfit t statistics (underfit). Third, the item PE3 (appropriate body position) showed misfit on the outfit t value (underfit) and last the item CF3 (cues patient before touching) showed inadequate fit (underfit) with regard to the outfit mean-square value. These four items were removed, and 25 items remained within the pool of items. From the 25 remaining items 3 items showed an overfit to the Rasch model. These were P5 (the overall assessment of the preparation), KD4 (the overall assessment of the knowledge and decision making) and PE7 (the overall assessment of the procedure execution). Item fit statistics for the remaining items are presented in Table 2. In addition, the item fit of all items and their corresponding threshold values with regard to infit t - statistics is visualised in Fig. 1.

**Table 2:** Overview item fit statistics of the APSPT with 25 items

Item ID	Item	Location Rasch measure	Location Score 0–100	Infit t	Outfit t	Infit MSQ	Outfit MSQ
P1	Plans procedure with regard to patient factors	0.75	38.73	−0.24	−0.37	0.95	0.92
P3	Adequate assessment is performed before the procedure	1.45	43.58	0.74	0.64	1.12	1.11
P4	Prepares patient appropriately	2.76	52.64	0.61	0.35	1.1	1.06
P5	Overall assessment preparation	1.34	42.81	−1.93	−2.07*	0.69	0.64
KD1	Shows knowledge of the procedure	1.39	43.16	−1.61	− 1.08	0.73	0.8
KD2	Shows knowledge of the steps of the procedure	1.79	45.93	−1.97	− 1.36	0.67	0.73
KD3	Identifies appropriate procedure	−0.55	29.73	−0.72	−0.17	0.86	0.73
KD4	Overall assessment knowledge	1.5	43.92	−2.85*	−2.52*	0.57	0.56
S1	Ensures other’s safety	2.34	49.74	1.47	1.07	1.29	1.26
S2	Ensures own safety	0.01	33.61	−0.06	0.2	0.97	1
S3	Overall assessment safety	−0.22	32.02	1.09	0.86	1.21	1.5
C1	Provides information about procedure	0.18	34.79	0.35	0.07	1.05	1
C2	Tells the patient to state if there is any pain or discomfort	3.16	55.41	1.37	1.46	1.3	1.48
C3	Communication during procedure	2.36	49.87	1.86	1.51	1.39	1.36
C4	Avoids jargon	2.19	48.7	0.91	0.13	1.19	0.85
C5	Asks if the patient has any questions	6.71	79.98	0.41	0.71	1.08	1.4
C6	Overall assessment communication	2.5	50.84	0.49	0.06	1.09	0.98
PE1	Appropriate hand and finger placement	1.54	44.2	1.38	1.38	1.25	1.31
PE2	Performs procedure correctly	2.31	49.53	−1.8	−1.51	0.69	0.71
PE4	Anticipates next step	1.25	42.19	−0.7	0.15	0.88	1.02
PE6	Appropriately adapts procedure to the patient	2.5	50.84	−1.48	−1.24	0.75	0.75
PE7	Overall assessment procedure execution	2.58	51.4	−3.39*	−3.03*	0.47*	0.41*
CF1	Appropriate patient positioning	2.41	50.22	0.1	−0.19	1.01	0.95
CF2	Responses to patient discomfort	1.97	47.18	−0.25	−0.37	0.94	0.88
CF4	Overall assessment comfort	1.99	47.31	−1.16	−1.06	0.78	0.73

NB**.** *indicates items showing overfit; the locations using the Rasch measure are reported in logit units (log-odds); the Location score is a transformation of the logit units into a 0–100 score. Higher scores and logits indicate higher item difficulties. The following abbreviations were used for the Item IDs: *P*: Preparation, *KD:* Knowledge and decision-making, *S:* Safety, *C:* Communication, *PE:* Procedure execution, *CF:* Comfort

### Threshold disordering and targeting

**Fig. 2:**
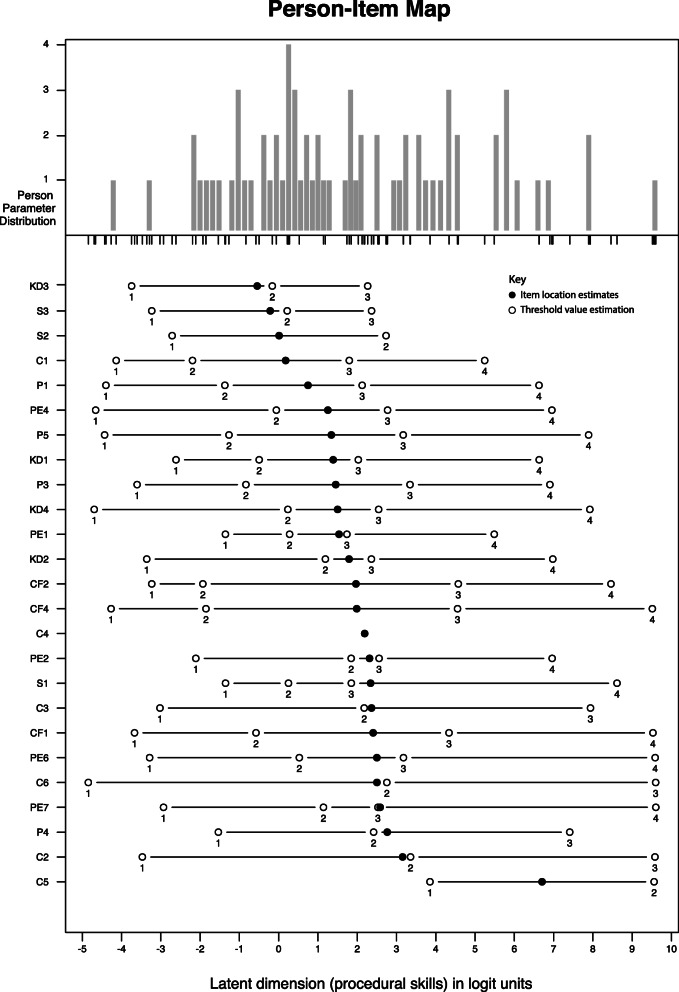
Person item map. The top part of the plot consists of a histogram of person abilities. Below the location of the item and item thresholds are plotted. Both abilities and difficulties are plotted along the latent dimension (procedural skills) with the unit logits

The person abilities ranged between − 4.21 and 9.58 logits (i.e. 4.38–99.85 on the 0–100 score). The item with the lowest difficulty estimate was item KD3 (Identifies appropriate procedure) -0.55 logits (29.76 on the 0–100 score) and the item with the highest difficulty estimate was C5 (listen to the patient and corresponding complaints) 6.71 logits (79.95 on the 0–100 score). Item thresholds ranged between − 4.85 logits for item C6 threshold 1 (0 on the 0–100 score) and 9.6 logits for item PE7 threshold 4 (100 on the 0–100 score). An overview of the location of the items and threshold parameters as well as the distribution of person parameters along the latent dimension is presented in Fig. 2. Targeting of the APSPT was adequate to measure the abilities of the included participants (i.e. the abilities of the participants were within the range of item thresholds). A tabulated overview of the threshold values is presented as Additional file 3. In addition, analysis of item thresholds showed that none of the items had disordered item thresholds. That is, threshold 1 was always less difficult than threshold 2, which was less difficult than threshold 3 and so on. However, one item C4 (avoids jargon) had only one threshold. That is all participants scored either 3 or 4 points and therefore there are not more threshold values available for this item.

The following example is given to provide an example of the parameter estimates. A general assumption in Rasch analysis is that if the difficulty estimate of an item and the estimate of the person’s abilities are equal, the person has a 50% chance of passing that item [36]. In our study, participant P7 had an ability estimate of 1.14 logits on the Rasch scale (i.e. this corresponds to 41.46 on the transformed 0–100 scale). An item with a similar difficulty estimate was the threshold 2 of item PE7 (i.e. 1.14 logits or 41.43 points). Therefore, participant P7 had a 50% chance to pass threshold 2, which was a rating of either 1 or 2 points.

### Goodness of fit of the Rasch model

Three tests were used to evaluate the goodness of fit of the APSPT to the Rasch model. First the Andersen LR-test indicated stability of item parameters over different groups of participants. As split criterion the performed procedure was used. That is the dataset was split into a group with the procedure “transfer” and the second group consisted of data from the procedure “vestibular rehabilitation”. An LR-value of 15.42 and the corresponding *p*-value of 0.422 indicated that the item functioning was similar between these groups. A second Anderson LR test was performed with the mean used as split criterion. That is one group consisted of participants scoring above the mean values and the other group consisted of participants scoring below the mean value. An LR value of 5.372 with a *p*-value of 0.147 showed item stability over these two groups.

**Fig. 3:**
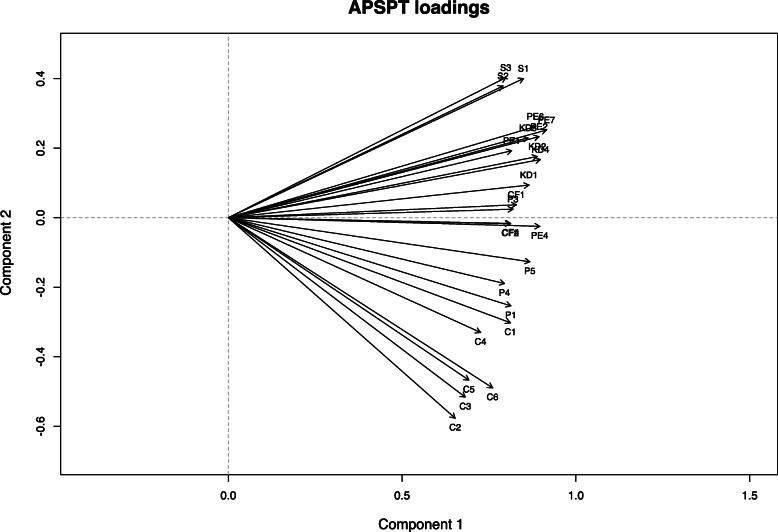
Plot showing the APSPT loadings in a two-dimensional space. Undimensionality is indicated when factor loadings point approximately in the same direction

Undimensionality was assessed using the Martin Löf test and categorical principal component analysis. Regarding the Martin Löf test an LR-value of 157 and the corresponding *p*-value of 0.99 indicated undimensionality. In addition, an exact version of the Martin Löf test (i.e. a nonparametric Rasch model tests for small samples) [34] showed an LR value of 60.78 with an exact p-value of 0.46 and confirmed undimensionality of the APSPT. Categorical principal component analysis was used to visually assess undimensionality. Factor loadings in a two-dimensional space pointed relatively homogenous in the same direction (Fig. 3). However, items of the “safety” and the “communication” subcategories deviated slightly from the loadings of the remaining items.

## Discussion

### Discussion of main results

This measurement properties study presented evidence for the structural validity of the APSPT in undergraduate education. First, item fit statistics showed reasonable fit of 25 items to the partial credit model (PCM) model. This indicates that all individual items provide information about the latent dimension “procedural skills”. Second, the tests of goodness of fit indicated that the assumptions of undimensionality and subgroup homogeneity were not violated. Therefore, it can be assumed that the overall score of the APSPT refers to the latent dimension “procedural skills”. Third, the difficulty parameters of the individual items and threshold values showed that it was possible to measure a broad capacity spectrum of participants in pre-registration physiotherapy with the APSPT.

Regarding the distribution of difficulties most sub-categories of procedural skills consisted of items with low difficulty locations and items with a moderate to high difficulty locations. This indicates that each sub-category covers the ability to measure a broad range of abilities. However, two categories had a different pattern of difficulty locations. First, items of the sub-category of “knowledge and decision-making” were located within the lower half of difficulty estimations (i.e. it was relatively easy to adequately score these items). This might be related to restrictions of this study. Participants had to choose from a limited number of options. The participants performing the procedure from vestibular rehabilitation knew that they had to perform a procedure targeted to treat a patient with vertigo and this considerably reduces the chance to select a non-adequate procedure. Similarly, the participants performing the transfer knew that they had to perform a transfer to ground. Therefore, the challenge to identify the appropriate procedure was lower than in real clinical situations. However, they still had to show knowledge about the procedure and steps of the procedure. In clinical situations where only limited prior knowledge is available it might be possible that the difficulty locations of these items might be different.

Analysis of the sub-category “communication” showed that most items were relatively difficult. With the exception of item C1 (provides information about the procedure) all items were located in the higher half of difficulty estimations. This might be because participants were all pre-registration students with limited patients encounters during clinical placements. Therefore, the ability to communicate adequately with a simulated patient might be challenging.

Distribution of the difficulty estimations of the “safety” sub-category showed that taking care of the patient safety was relatively easy. However, the ability to ensure one’s own safety was more challenging. This might indicate that the participants prioritised patient safety during execution of the procedure and it was more difficult to ensure personal safety. This represents a challenge for educators and should be targeted during education of procedures.

Recently Judd and colleagues [37] have reported on the validity of the Assessment of Physiotherapy Practice (APP), which is a professional competence tool for physiotherapy students in simulation based clinical. The latent dimension of this tool (i.e. professional competence) is relatively broad compared to the latent dimension of the APSPT, which focuses on the performance of procedural skills. Procedural skills are a part of the professional competence and therefore both assessments measure partly similar information. For example, the category “intervention” of the APP contains items such as “performs intervention appropriately”, which is very similar to the item “performs procedure correctly” of the APSPT. However, other items of the APP such as “demonstrate commitment to learning” are not within the scope of the APSPT. Similar to this study the APP has been validated using Rasch analysis and the authors have reported adequate structural validity of the APP [37]. Future studies might set out to test the correlation between the APSPT and the APP’s subscale of procedural skills to further validate the APSPT.

McKinley et al. [9] proposed in their systematic review that several subcategories or themes should be integrated into generic assessment tools for procedural skills (e.g. items related to safety or communication). The APSPT was designed as generic assessment tool and therefore consisted of several subcategories. However, one assumption was that all subcategories provide relevant information about the latent dimension “procedural skills”. Only when this assumption is valid the total score of the APSPT can be used as a measure of the latent dimension [14]. Several goodness of fit indices indicated undimensionality and therefore we propose that the summary score of the APSPT is a valid measure of procedural skills. However, categorical principal component analysis showed that the factor loadings of the subcategories “safety” and “communication” were at the opposite ends of the latent dimension. That is arguably both subcategories provide relevant information but the remaining subcategories such as “preparation”, “knowledge and decision-making”, “procedure execution” and “comfort” are central parts of the latent dimension. A short version of the APSPT might be designed in future studies consisting only of the latter subcategories.

Three items with indications of overfit remained in the item pool. However, two items showed no misfit on the mean-square fit indices. These items were not removed in order to avoid a type I error. One item with overfit on all four fit statistics remained in the item pool. However, the mean-square values (0.47 and 0.41) were only slightly below the threshold of 0.5. Furthermore, overfit indicated that the item was less productive for the measurement, but did not degrade the measurement [30]. All three items with overfit were “overall assessments” of a specific sub-category (i.e. preparation, knowledge and decision-making and procedure execution), which might explain the overfit because part of the information used to score these items was also used to score specific items of the corresponding sub-category. For example, information used to evaluate the specific item PE1 (appropriate hand and finger placement) may also be used to evaluate item PE7 (overall assessment procedure execution).

### Limitations

The APSPT was validated using students from undergraduate education within a 3-year bachelor programme in Switzerland. This a specific group and therefore, the APSPT can be used to evaluate procedural skills in this or a similar group. However, it is possible that the measurement properties of the APSPT change when students from different educational programmes (such as post-graduate education programmes) or countries with a different structure of their physiotherapy education are evaluated.

A further limitation of this measurement study was the relatively small sample size. Using small sample sizes produces less precise and robust estimates and a less powerful fit analysis. With the current sample size the locations of the person and item parameters are stable within + − ½ logits with 95% confidence [29]. Therefore, the findings of this study provide relevant information about the structural validity of the APSPT but it is possible that future studies with larger sample sizes can change the parameters estimated with the Rasch model.

Regarding the analysis, Smith et al. [38] reported that mean-square fit statistics are less sample size dependent than t-statistics, which were found to increase type I error rates (i.e. falsely rejecting an item as not fitting). Therefore, t-statistics were analysed with caution.

The APSPT was designed as a generic assessment tool for procedural skills in physiotherapy education. Within this study the APSPT was used to evaluate two procedures. Both procedures were from different areas of physiotherapy practice (neurological physiotherapy and vestibular rehabilitation) but future studies should set out to explore the applicability of the APSPT to other areas such as musculoskeletal practice as well.

### Implications

The results of this study apply to the evaluation of undergraduate physiotherapy students (i.e. in the context of a 3-year bachelor’s degree course) and to institutions with a similar physiotherapy programme as the HES-SO Valais-Wallis and to the country Switzerland.

This measurement properties study has several implications for educational practice. First, the APSPT is a validated generic tool to measure procedural skills in physiotherapy education. Relatively few assessments for procedural skill exist to measure this latent dimension and therefore educators can use this assessment tool to measure the effectiveness of their educational intervention.

Furthermore, educators might use the APSPT to target their teaching to the individual student. For example, by identification of problems within specific subcategories or by taking into account the difficulties of the individual items. In addition, the abilities of the students can be compared on a linear interval scaled continuum.

The implications for research include that the findings of this study should be controlled for in a study with a larger sample size to increase the precision of the estimated parameters of the Rasch analysis. In addition, the APSPT should be validated within different educational levels (e.g. MSc students), students with different amounts of previous experience (i.e. ranging from complete novices to experts), different countries and their educational systems. This information is essential before the evidence of this study can be generalised to other institutions, physiotherapy degree programmes or countries.

The findings of this study can be used to create a computer adaptive test of the APSPT, which must be validated in a future study. Then, there is a need for more information about other measurement properties of the APSPT such as construct validity. Finally, future studies might set out to design a short form of the APSPT.

## Conclusion

This study presented evidence for the structural validity of the APSPT. Undimensionality of the APSPT was confirmed and therefore presents evidence that the latent dimension of procedural skills in physiotherapy education consists of several subcategories. A generic assessment of procedural skills should therefore be based not only on the evaluation of biomechanical aspect but also on aspects related to preparation, safety, knowledge and decision making, communication and comfort. However, the findings should be interpreted with caution regarding caveats such as the limited sample size.

## Supplementary information

**Additional file 1.** APSPT with 29 items. The APSPT with 29 items used in the current study.

**Additional file 2.** Modified_STROBE_checklist.doc. Checklist containing the location of reported STROBE items.

**Additional file 3.** APSPT Item and threshold locations. The estimated logit value and transformed score for each item and all thresholds.

## Data Availability

The datasets analysed during the current study are available from the corresponding author on reasonable request.

## Abbreviations

APSPT: Assessment of procedural skills in physiotherapy education
