# Supplementary material for: The assessment of procedural skills in physiotherapy education: a measurement study using the Rasch model
Source: Arch Physiother. 2020 May 25;10:9. doi: 10.1186/s40945-020-00080-0 (PMC7249622; doi:10.1186/s40945-020-00080-0)
Supplement: Supplementary file 1 — Additional file 1. APSPT with 29 items. The APSPT with 29 items used in the current study. [file 40945_2020_80_MOESM1_ESM.docx]

## Additional file 1: APSPT with 29 items

| **ID** | **Item** | **Evaluation** | **Remarks** |
| --- | --- | --- | --- |
| **Preparation** | | | |
| P1 | Plans procedure with regard to patient factors |  |  |
| P2 | Checks and prepares environment |  |  |
| P3 | Adequate assessment is performed before the procedure |  |  |
| P4 | Prepares patient appropriately |  |  |
| P5 | **Overall assessment preparation** |  |  |
| **Knowledge & decision making** | | | |
| KD1 | Shows knowledge of the procedure |  |  |
| KD2 | Shows knowledge of the steps of the procedure |  |  |
| KD3 | Identifies appropriate procedure |  |  |
| KD4 | **Overall assessment knowledge** |  |  |
| **Safety** | | | |
| S1 | Ensures other's safety |  |  |
| S2 | Ensures own safety |  |  |
| S3 | **Overall assessment safety** |  |  |
| **Communication** | | | |
| C1 | Provides information about procedure |  |  |
| C2 | Tells the patient to state if there is any pain or discomfort |  |  |
| C3 | Communication during procedure |  |  |
| C4 | Avoids jargon |  |  |
| C5 | Asks if the patient has any questions |  |  |
| C6 | **Overall assessment communication** |  |  |
| **Procedure execution** | | | |
| PE1 | Appropriate hand and finger placement |  |  |
| PE2 | Performs procedure correctly |  |  |
| PE3 | Appropriate body position |  |  |
| PE4 | Anticipates next step |  |  |
| PE5 | Uninterrupted flow of the procedure |  |  |
| PE6 | Appropriately adapts procedure to the patient |  |  |
| PE7 | **Overall assessment procedure execution** |  |  |
| **Comfort** | | | |
| CF1 | Appropriate patient positioning |  |  |
| CF2 | Responses to patient discomfort |  |  |
| CF3 | Cues patient before touching |  |  |
| CF4 | **Overall assessment comfort** |  |  |
