# Supplementary material for: The assessment of procedural skills in physiotherapy education: a measurement study using the Rasch model
Source: Arch Physiother. 2020 May 25;10:9. doi: 10.1186/s40945-020-00080-0 (PMC7249622; doi:10.1186/s40945-020-00080-0)
Supplement: Supplementary file 2 — Additional file 2. Modified_STROBE_checklist.doc. Checklist containing the location of reported STROBE items. [file 40945_2020_80_MOESM2_ESM.docx]

## Modified STROBE – checklist for observational studies

based on Cook et al. (1))

| Item No | Strobe Recommendation | Coded Element (Cook et al.) | **Operational definition (Cook et al.)** | Reported on page # |
| --- | --- | --- | --- | --- |
| **Introduction** | | | |  |
| 1a | (*a*) Indicate the study’s design with a commonly used term in the title or the abstract | Study design in title | An explicit statement of study design is present in the title. We counted explicit or implied comparisons as "vague" unless accompanied by an explicit study design. The word "evaluation" did not count as a study design. | 1 |
| 1b | (*b*) Provide in the abstract an informative and balanced summary of what was done and what was found | Rationale | The rationale (variably termed background or context) describes the impetus for the project, often taking the form of a description of existing knowledge on the topic or a problem statement. | 2 |
| 1b |  | Objective | The objective clearly states the intent of the study. | 2 |
| 1a |  | Study design (in abstract) | An explicit and accurate statement identifies the nature of the study design. | 2 |
| 1b |  | Setting | The setting describes the educational context of the study in a way that allows the reader to determine whether results apply in their own circumstances. Either general terms or institution name will suffice. | 2 |
| 1b |  | Participants (stage of training) | The stage of training of study participants is clearly identified. We required that this include the specific year of training unless the participants were in practice. | 2 |
| 1b |  | Participants (number) | The number of study participants is clearly identified. | 2 |
| 1b |  | Nature of intervention | The definition of the study intervention provides a basic understanding of what happened. | n.a. |
| 1b |  | Nature of control/comparison | The definition of the comparison intervention or control group provides a basic understanding of what happened to this group. | n.a. |
| 1b |  | Outcome measures | The main study outcome variables (e.g. cardiac auscultation skill) or instruments (e.g. OSCE in cardiac auscultation) are indicated. | 2 |
| 1b |  | Results: data presented | The main results are reported. Results presenting quantitative or qualitative data were distinguished from results that presented only a narrative summary of findings. Statistical test results alone (e.g., p values) were insufficient. | 2 |
| 1b |  | Conclusions | The conclusions provide a brief interpretation of study results. | 2 |
| **Methods** | | | | |
| 2 | Background/rationale | Critical literature review | The literature review cites articles relevant to the topic (e.g., prior approaches to teaching this topic) or instructional design (e.g., Internet-based instruction), and critically discusses these articles at the beginning of the paper. | 3-4 |
| 2 | Background/rationale | Conceptual framework | The conceptual framework situates the research question, intervention methods, or study design within a model or theoretical framework that facilitates meaningful interpretation of the methods and results. | 4-5 |
| 2 | Background/rationale | Problem statement | The problem statement clearly identifies the gap between what is known on this topic (e.g. previously defined empiric evidence or theory) and what is not known. | 4-5 |
| 3 | State specific objectives, including any prespecified hypotheses | Statement of study intent | Study intent can be phrased as a research question, a research hypothesis, or a purpose (goal, aim). | 5 |
| 4 | Present key elements of study design early in the paper | Explicit statement of study design | An explicit and accurate statement identifies the nature of the study design. | 6 |
| 5 | Describe the setting, locations, and relevant dates, including periods of recruitment, exposure, follow-up, and data collection | Location | The study location clearly indicates the precise institution[s] or region (city or county) in which the study took place. Web-only interventions should specify a home institution. | 6 |
| 5 |  | Dates | The report clearly indicates the year in which the study (educational intervention) occurred. This must include at least one date (beginning or end date) and either the other date or the duration of the study. | 6 |
| 6 | Participants: Give the eligibility criteria, and the sources and methods of selection of participants. | Eligibility criteria | The report contains an explicit statement indicating who was eligible to participate in the study. | 6 |
| 6 |  | Method for selection/enrollment | The report contains an explicit statement indicating how participants were selected. This is different than eligibility: eligibility concerns who could have been included, while selection indicates how people were actually included in the study. | 6 |
| 7 | Clearly define all outcomes, exposures, predictors, potential confounders, and effect modifiers. | Intervention defined | The study intervention is described in sufficient detail as to permit replication. | n.a. (this was a measurement properties study. No intervention was performed) |
| 7 |  | Comparison group defined | The comparison intervention or control group is described in sufficient detail as to permit replication. Comparisons with no intervention should clearly state that this is the case. | n.a. (this was a measurement properties study. No intervention was performed) |
| 7 |  | Major outcome(s) defined in conceptual terms. | The outcome(s) is defined using an explicit conceptual term such as knowledge, communication skill, influenza vaccination rate, etc. | 7 |
| 8 | For each variable of interest, give sources of data and details of methods of assessment (measurement). | Description of outcome | The report clearly states how the primary outcome was measured, and provides some detail about the instrument or method (e.g., the number of stations and the rating form in an OSCE, the number and type of questions in a MCQ test, etc). | *7* |
| 9 | Describe any efforts to address potential sources of bias | Description of measures employed to control bias in selection (sampling, group assignment) | The report describes efforts made to ensure a representative sample or equal distribution of participants between groups. These measures might include: enrolling a high percentage of eligible pool, random sampling of enrolled participants from a larger eligible pool, describing the method of randomization, providing other details on group assignment methods, matching or adjusting for key covariates, and minimizing bias between groups at the end of the study (e.g. promoting high follow-up rate). | 6 |
| 9 |  | Description of measures employed to control bias in information (exposure, outcome assessment) | The report describes efforts made to ensure that the exposures and assessments were of similar quality between the groups. These measures might include: verifying that both groups received the interventions as planned, reporting unexpected deviations from the planned interventions, using the same assessment instrument for both groups, and avoiding a specific type of assessment that would unfairly advantage one group. | n.a. |
| 10 | Explain how the study size was arrived at | Sample size calculation | A sample size calculation is reported. We did not count post- hoc calculations or justification of sample size in the Discussion section. | 7 |
| 11 | Explain how quantitative variables were handled in the analyses. If applicable, describe which groupings were chosen and why | Decisions about data handling (cutpoints, modeling assumptions) | The report describes cutpoints, modeling assumptions, or other details of quantitative data handling. | 7-8 |
| 12 | (*a*) Describe all statistical methods, including those used to control for confounding | Statistical methods described. | The report describes the statistical methods in sufficient detail to judge appropriateness and permit replication. | 7-8 |
| **Results** | | | | |
| 13a | Report numbers of individuals at each stage of study | Number eligible | The report states the exact number eligible. The numbers can be reported in the main text, or in a table or figure. | 9 |
| 13a |  | Number enrolled | The report states the exact number enrolled. | 9 |
| 13a |  | Number complete follow-up | The report states the exact number providing data at the end of the study. | 9 |
| 13b | Give reasons for non- participation at each stage | Reasons for non- participation provided | The report states (at least briefly) the reasons for non- participation for all eligible participants who declined an invitation to enroll. | 9 |
| 13c | Consider use of a flow diagram | Flow diagram present | A flow diagram is present in the main text or as an appendix. | A flow diagram was not included because only a single measurement point was used in this study. |
| 14 | Give characteristics of study participants | Training level | The report notes the training level of participants in each study. This can be done in the main text or in a table or figure. Training level must include specific year of training until in independent practice, and those in practice should be identified as such. | 9 (Table 1) |
| 14 |  | Other demographics | The report describes other demographic characteristics including but not limited to gender, race, age, learning style, etc. | 9 (Table 1) |
| 15 | Report numbers of outcome events or summary measures over time | Mean and measure of spread, or exact count or percentage with denominator | For continuous variables, the report presents both the mean and a measure of variance (e.g., standard deviation, standard error of the mean). A confidence interval for the mean would suffice if the sample size in that analysis is clear. | 10-11 |
| 16 | Give unadjusted estimates and, if applicable, confounder-adjusted estimates and their precision (eg, 95% confidence interval). Make clear which confounders were adjusted for and why they were included | p value (must be exact if p>.05) | The report presents p values for each major analysis. We coded this as "present" if the information was provided for at least one major outcome. Although exact p values were preferred, for p<.05 (or some other level of alpha) we accepted (coded as "present") “p<[alpha]” since journal or field conventions often dictate such reporting. However, for all p>alpha we required provision of an exact p value. | 10-11 |
| 16 |  | Confidence interval for difference in means or ratio | For continuous variables, the report presents the confidence interval for the difference in means. For categorical variables, the report presents the confidence interval for the ratio. | 10-11 |
| 17 | Report other analyses done | Subgroup and sensitivity analyses identified a priori | If subgroup analyses are reported, the report should make it clear (i.e. in the Methods) that these were planned rather than post hoc. | n.a. |
| **Discussion** | | | | |
| 18 | Summarise key results with reference to study obbjectives | Summarize key results with reference to objectives | The report includes both a brief summary of main findings and a statement of how these findings inform or answer the study objective/hypothesis/question. Given the wording of the STROBE recommendation, we counted summaries without reference to study objectives as "vague." | 12 |
| 19 | Discuss limitations of the study, taking into account sources of potential bias or imprecision | Limitations / strengths – sources of bias | The report describes potential limitations or strengths of the study as regards sources of bias (e.g., unequal group assignment, incomplete follow-up, assessment measures that might favor one group over the other). | 15 |
| 19 |  | Limitations / strengths – precision (sample size, power) | The report describes potential limitations or strengths of the study as regards precision (e.g., power, sample size, confidence intervals in relation to a desired effect size). | 15 |
| 19 |  | Limitations / strengths – educational significance (magnitude of effect, confounding) | The report describes potential limitations or strengths of the study as regards the educational or clinical significance of results (e.g. Is the effect size educationally significant? Was confounding present? Were outcomes educationally meaningful?). |  |
| 19 | Give a cautious overall interpretation of results considering objectives,  limitations, multiplicity of analyses, results from similar studies, and other relevant evidence | Integration with other studies and/or theory | The report contrasts results with previous studies. This requires more than simply citing the previous work; the present study must also be held up for comparison followed by a re-interpretation of the literature in light of the present body of evidence. | 13-14 |
|  |  | Interpretation in light of limitations | There is an explicit attempt to couch conclusions in light of the limitations noted above. | 15 |
|  | Discuss the generalisability (external validity) of the study results | Generalizability (application to practice and/or relevance to future research) | There is an explicit attempt to frame the scope or application of these results for educational practice or future research. | 16 |
| **Other elements** | | | | |
|  | Give the source of funding | Funding source identified | The funding source is identified somewhere in the report. | 17 |
|  | Non-STROBE: Attention to Human Subject Protections | Institutional Review Board review noted | Approval or exemption from an Institutional Review Board is noted, or note is made that such review is not required per local policy. | 6 |
|  |  | Participant consent noted Participant consent | Participant consent is noted. | 6 |

## References

1. Cook DA, Levinson AJ, Garside S. Method and reporting quality in health professions education research: a systematic review. Med Educ. 2011;45(3):227-38.
