# Supplementary material for: The assessment of procedural skills in physiotherapy education: a measurement study using the Rasch model
Source: Arch Physiother. 2020 May 25;10:9. doi: 10.1186/s40945-020-00080-0 (PMC7249622; doi:10.1186/s40945-020-00080-0)
Supplement: Supplementary file 3 — Additional file 3. APSPT Item and threshold locations. The estimated logit value and transformed score for each item and all thresholds. [file 40945_2020_80_MOESM3_ESM.docx]

## Additional file 3: APSPT Item and threshold locations

| **ID** | **Location Rasch** | **Threshold 1 Rasch** | **Threshold 2 Rasch** | **Threshold 3 Rasch** | **Threshold 4 Rasch** | **Location Score 0-100** | **Threshold 1 Score 0-100** | **Threshold 2 Score 0-100** | **Threshold 3 Score 0-100** | **Threshold 4 Score 0-100** |
| --- | --- | --- | --- | --- | --- | --- | --- | --- | --- | --- |
| P1 | 0.75 | -4.4 | -1.37 | 2.13 | 6.63 | 38.71 | 3.1 | 24.06 | 48.26 | 79.42 |
| P3 | 1.45 | -3.6 | -0.83 | 3.34 | 6.91 | 43.6 | 8.6 | 27.77 | 56.69 | 81.34 |
| P4 | 2.76 | -1.54 | 2.42 | 7.41 | NA | 52.67 | 22.92 | 50.27 | 84.83 | NA |
| P5 | 1.34 | -4.43 | -1.26 | 3.17 | 7.89 | 42.82 | 2.87 | 24.79 | 55.49 | 88.15 |
| KD1 | 1.39 | -2.61 | -0.5 | 2.03 | 6.63 | 43.15 | 15.45 | 30.11 | 47.58 | 79.45 |
| KD2 | 1.79 | -3.36 | 1.19 | 2.36 | 6.98 | 45.95 | 10.29 | 41.77 | 49.88 | 81.85 |
| KD3 | -0.55 | -3.74 | -0.16 | 2.27 | NA | 29.76 | 7.62 | 32.42 | 49.23 | NA |
| KD4 | 1.5 | -4.7 | 0.23 | 2.54 | 7.92 | 43.93 | 1.04 | 35.16 | 51.15 | 88.38 |
| S1 | 2.34 | -1.35 | 0.25 | 1.85 | 8.61 | 49.74 | 24.18 | 35.27 | 46.34 | 93.16 |
| S2 | 0.01 | -2.71 | 2.73 | NA | NA | 33.62 | 14.77 | 52.46 | NA | NA |
| S3 | -0.22 | -3.23 | 0.22 | 2.37 | NA | 32.05 | 11.18 | 35.05 | 49.92 | NA |
| C1 | 0.18 | -4.14 | -2.19 | 1.8 | 5.25 | 34.77 | 4.91 | 18.36 | 45.99 | 69.84 |
| C2 | 3.16 | -3.47 | 3.36 | 9.58 | NA | 55.38 | 9.52 | 56.78 | 99.85 | NA |
| C3 | 2.36 | -3.02 | 2.18 | 7.94 | NA | 49.91 | 12.63 | 48.61 | 88.48 | NA |
| C4 | 2.19 | 2.19 | NA | NA | NA | 48.7 | 48.7 | NA | NA | NA |
| C5 | 6.71 | 3.85 | 9.56 | NA | NA | 79.95 | 60.2 | 99.7 | NA | NA |
| C6 | 2.5 | -4.85 | 2.75 | 9.6 | NA | 50.86 | 0 | 52.6 | 99.97 | NA |
| PE1 | 1.54 | -1.36 | 0.28 | 1.74 | 5.49 | 44.18 | 24.15 | 35.47 | 45.57 | 71.53 |
| PE2 | 2.31 | -2.11 | 1.85 | 2.56 | 6.96 | 49.56 | 18.94 | 46.32 | 51.24 | 81.74 |
| PE4 | 1.25 | -4.66 | -0.06 | 2.77 | 6.96 | 42.21 | 1.29 | 33.13 | 52.73 | 81.69 |
| PE6 | 2.5 | -3.29 | 0.53 | 3.18 | 9.59 | 50.85 | 10.8 | 37.18 | 55.53 | 99.88 |
| PE7 | 2.58 | -2.93 | 1.14 | 2.53 | 9.6 | 51.43 | 13.25 | 41.43 | 51.03 | 100 |
| CF1 | 2.41 | -3.67 | -0.58 | 4.34 | 9.53 | 50.19 | 8.14 | 29.55 | 63.56 | 99.5 |
| CF2 | 1.97 | -3.23 | -1.93 | 4.57 | 8.46 | 47.16 | 11.18 | 20.18 | 65.2 | 92.1 |
| CF4 | 1.99 | -4.27 | -1.85 | 4.55 | 9.51 | 47.3 | 4.01 | 20.73 | 65.06 | 99.39 |

**Nb.** Location of items and item thresholds are presented. The locations using the Rasch measure are reported in logit units (log-odds); the location score is a transformation of the logit units into a score of 0-100 In addition, threshold locations are reported (i.e. in logits for the Rasch measure and transformed to a 0-100 score)
